# Historical Perspectives on the Evolution of Stressor Research

**DOI:** 10.1093/geroni/igaf122.933

**Published:** 2025-12-31

**Authors:** David Chiriboga

**Affiliations:** University of South Florida, Tampa, Florida, United States

## Abstract

This presentation will focus on changes in how stressors, the things that cause distress, have been studied. Early work was generally of two types (1): lab studies where people, often students, were exposed to stressful film, electric shock, or stressing situations; (2) studies of how people responded to natural disasters, bereavement, etc. These studies primarily focused on post-stress reactions, and on how people coped. During the 60s the development of inventories of potentially stressful events broadened the scope of stress research to include so-called “life events”. Several inventories were developed, with the Holmes and Rahe (1967) Schedule of Recent Events the best known. However, a number of inventories were developed, with several including events in later life. Underlying this second wave of research was the idea that how people perceived the stressful nature of events was associated with outcomes, although there was an initial, erroneous assumption that most people agree about how much disruption the various events caused. In addition to this research, there were a number of researchers who looked at what might be called micro events (e.g., daily hassles), or macro events like economic downturns or the threat of war. While for many years there was general agreement about the adequacy of specific measures, their use became less consistent over the past two decades. Today, the approach to stressor measurement is much more varied, with little consensus as to which is the most effective. Overall, however, stressors continue to demonstrate their relevance to the study of behavioral outcomes.

